# Value drivers and barriers to the adoption of Precision Psychiatry in Latin America: a qualitative study

**DOI:** 10.1186/s12888-026-08038-5

**Published:** 2026-04-03

**Authors:** Margarita Quijano, João Victor Rocha, Hilary Felton, Jesús Ramírez-Bermúdez, Humberto Correa, Carlos Lopez Jaramillo, Alessandro Serretti, Eduard Vieta

**Affiliations:** 1 Policy Wisdom LLC, Puerto Rico, USA; 2 Neuropsychiatry Unit, National Institute of Neurology and Neurosurgery, Mexico City, Mexico; 3 Department of Mental Health, Faculdade de Medicina, Universidade Federal de Minas Gerais, Belo Horizonte, Brazil; 4 Department of Psychiatry, School of Medicine, Universidad de Antioquia, Medellin, Colombia; 5 Department of Medicine and Surgery, Kore University of Enna, Enna, Italy; 6 Institute of Neuroscience, University of Barcelona, Hospital Clinic, IDIBAPS, CIBERSAM-ISCIII, Barcelona, Catalonia, Spain

**Keywords:** Precision medicine, Precision psychiatry, Major depressive disorders, Qualitative study, Latin America

## Abstract

**Background:**

Treating major depressive disorder is difficult due to the lack of objective criteria to guide therapy. Decision making often relies on trial and error, which limits treatment effectiveness. Precision psychiatry has been presented as a new approach that uses biological and multimodal data to support diagnosis and treatment, similar to precision medicine in other fields.

**Aims:**

This study aims to explore the perceptions of key experts regarding the drivers and barriers to the adoption of precision psychiatry in Latin America. Secondary objectives are to explore the conceptualization of precision psychiatry, explore its potential benefits in the context of MDD, identify potential drivers and barriers to its implementation in the region, and identify context specific considerations for future implementation.

**Methods:**

This is a qualitative study based on interviews, and a hybrid deductive-inductive content analysis was applied. A scoping review informed the development of a semi structured interview guide. Semi structured interviews were then conducted with experts involved in healthcare provision, mental health policy shaping, and academic research. The interviews explored the perceived value of precision psychiatry, its potential contribution to the treatment of major depressive disorder, and the challenges surrounding its adoption in the region.

**Results:**

Participants viewed precision psychiatry as a promising approach to improve diagnostic accuracy, guide targeted treatment, and use healthcare resources more efficiently. These advantages were seen as particularly relevant for major depressive disorder due to its high burden, heterogeneity, and prevalence of treatment resistant cases in Latin America. Key barriers included limited understanding of precision concepts, insufficient evidence on effectiveness and cost, low provider awareness, high implementation costs, regulatory constraints, and systemic challenges such as under-resourced services, stigma, and shortages of trained professionals.

**Conclusion:**

Precision psychiatry has the potential to improve mental health care in Latin America according to participants, especially for major depressive disorder. Realizing this potential will require stronger evidence, greater investment in research and training, improved information systems, stronger mental health policies, and regulatory processes that can assess clinical, economic, and societal value. Coordinated efforts across stakeholders are necessary to support its development and adoption.

**Clinical trial number:**

Not applicable.

**Supplementary Information:**

The online version contains supplementary material available at 10.1186/s12888-026-08038-5.

## Introduction

Major depressive disorders (MDD) are among the leading causes of disability worldwide [1] and the eleventh leading cause of disability in the Region of the Americas, according to evidence from 2019 [2]. MDD causes severe functional impairment and adversely affects interpersonal relationships, lowering the quality of life of patients [1]. MDD has significant impact in Latin America (LATAM) countries regarding burden of disease, socioeconomic, and well-being dimensions. Regarding burden of disease, evidence indicates a high prevalence of treatment-resistant depression (TRD) in the region [3], higher proportion of suicide tendencies among patients with TRD in Argentina [4], higher MDD prevalence among women in Brazil [5], and depression often being untreated in Mexico and Peru [6, 7]. MDD has an impact on well-being, too, given that patients with TRD have more comorbidities and report greater work impairment in LATAM [3]. Country evidence from Argentina, Brazil, and Mexico also shows MDD and TRD leading to lower quality of life, both for patients and caregivers [8, 9]. MDD also has a significant socioeconomic impact in the region, with estimations from 2021 of lost contribution to economies due to mental disorders that lead to disability or death among young people at nearly US$30.6 billion a year in LATAM [10].

Treating MDD and its symptoms is challenging, given the absence of objective criteria to define treatment. Under the current model of care, healthcare providers must navigate the selection among various pharmacological options, usually making decisions on a trial-and-error basis [11], which ultimately leads to patients not receiving optimal care [12]. In response to this challenge, novel approaches have been developed, providing mechanisms of action that are more specific to neurobiological pathways connected to the clinical presentation of neurological and psychiatric diseases subtypes. Such advances hold the potential to improve patient care by providing targeted therapies tailored to the individual’s specific multidimensional characteristics, including through early screening, accurate detection of differentiated pathophysiological signatures, and preventative strategies [13]. For example, the use of pharmacogenetics may foster accuracy in treatment selection for MDD [14].

Emerging views suggest that precision in the treatment of MDD could help optimize the selection of treatment by incorporating biological and environmental data to enhance treatments and complement clinical judgement when prescribing antidepressants [11, 15]. Moreover, by integrating new information points of entry (coming from genetic testing and clinical assessment), it will also contribute to therapeutic drug monitoring [11, 16]. In this context, understanding the value drivers of precision psychiatry and the challenges to its implementation in LATAM, a necessary first step is to bring the benefits of a precision approach closer to MDD patients, providers, and the health system as a whole.

The new field of “Precision Psychiatry” is gaining momentum since its first description in 2015 [17], and it describes an application of precision medicine to the field of psychiatry. Precision Medicine is defined by the US National Human Genome Research Institute as an innovative approach that uses information about an individual’s genomic, environmental, and lifestyle information to guide decisions related to their medical management. The goal of precision medicine is to provide a more precise approach for the prevention, diagnosis, and treatment of disease [18].

Precision psychiatry is presented in the literature as a paradigm shift in which psychiatric concepts and practices are based on the use of new technologies and the aggregation of data stemming from a multimodal assessment of patients [19]. Modeled on the successful application of precision medicine in other fields, the aim of precision psychiatry is to introduce innovative and objective methodologies for the diagnosis and treatment of mental health conditions [20, 21], which could lead to improved access to accurate diagnosis, prediction of response or non-response to treatment, and prognosis [19, 21–23].

Given that precision psychiatry is an emerging field characterized by a growing scientific literature but limited real world evidence, it is important to examine its potential benefits as well as the barriers to its implementation in anticipation of potential uptake. The primary aim of this study is to explore the perceptions of key experts regarding the drivers and barriers to the adoption of precision psychiatry in LATAM, particularly in the context of MDD. The secondary objectives were: (1) to examine how precision psychiatry is understood and positioned; (2) to explore experts’ views on the potential added value of precision psychiatry for patients, caregivers, and health systems, especially in the treatment of MDD; and (3) to identify context-specific considerations relevant to future implementation, including stakeholder involvement and system-level readiness. By focusing on expert perceptions within LATAM, this study aims to provide region-specific insights into the potential opportunities and challenges associated with implementing this emerging approach in the region.

## Methods

### Study design

This is a qualitative study based on semi-structured interviews with physicians with experience in defining and/or designing care pathways, protocols or other healthcare management roles to explore the concept, the value drivers, and the challenges of the adoption of precision psychiatry in LATAM, especially the treatment of MDD. The results are reported in accordance with the Consolidated criteria for Reporting Qualitative Research Checklist (COREQ) [24] as presented in Table 1.

**Table 1:** Consolidated criteria for reporting qualitative studies (COREQ): 32-item checklist

Item No	Guide Questions/Description	Reported on Section #
**Domain 1: Research team and reflexivity**		
**Personal Characteristics**		
1. Interviewer/ facilitator	Which author/s conducted the interview or focus group?	Section “Data collection”
2. Credentials	What were the researcher’s credentials? E.g., PhD, MD	Section “Research team and reflexivity”
3. Occupation	What was their occupation at the time of the study?	Section “Research team and reflexivity”
4. Gender	Was the researcher male or female?	N/A
5. Experience and training	What experience or training did the researcher have?	Section “Research team and reflexivity”
**Relationship with participants**		
6. Relationship established	Was a relationship established prior to study commencement?	Section “Research team and reflexivity”
7. Participant knowledge of the interviewer	What did the participants know about the researcher? e.g. personal goals, reasons for doing the research?	Section “Research team and reflexivity”
8. Interviewer characteristics	What characteristics were reported about the interviewer/facilitator? e.g. Bias, assumptions, reasons and interests in the research topic	Section “Research team and reflexivity”
**Domain 2: study design**		
**Theoretical framework**		
9. Methodological orientation and Theory	What methodological orientation was stated to underpin the study? e.g. grounded theory, discourse analysis, ethnography, phenomenology, content analysis	Section “Data analysis”
**Participant selection**		
10. Sampling	How were participants selected? e.g., purposive, convenience, consecutive, snowball	Section “Participants and recruitment”
11. Method of approach	How were participants approached? e.g., face-to-face, telephone, mail, email	Section “Participants and recruitment”
12. Sample size	How many participants were in the study?	Section “Participants and recruitment”
13. Non-participation Setting	How many people refused to participate or dropped out? Reasons?	Section “Participants and recruitment”
14. Setting of data collection	Where was the data collected? e.g., home, clinic, workplace	Section “Participants and recruitment”
15. Presence of nonparticipants	Was anyone else present besides the participants and researchers?	N/A
16. Description of sample	What are the important characteristics of the sample? e.g. demographic data, date	Suppl. Mat.
**Data collection**		
17. Interview guide	Were questions, prompts, and guides provided by the authors? Was it pilot tested?	Section “Study Design”
18. Repeat interviews	Were repeat interviews carried out? If yes, how many?	N/A
19. Audio/visual recording	Did the research use audio or visual recording to collect the data?	Section “Data collection”
20. Field notes	Were field notes made during and/or after the interview or focus group?	Section “Data collection”
21. Duration	What was the duration of the interviews or focus group?	Section “Data collection”
22. Data saturation	Was data saturation discussed?	Section “Data collection” and Section “Strengths and Limitations”
23. Transcripts returned	Were transcripts returned to participants for comment and/or correction?	N/A
**Domain 3: analysis and findings**		
**Data analysis**		
24. Number of data coders	How many data coders coded the data?	Section “Data analysis”
25. Description of the coding tree	Did the authors provide a description of the coding tree?	N/A
26. Derivation of themes	Were themes identified in advance or derived from the data?	Section “Data analysis”
27. Software	What software, if applicable, was used to manage the data?	Section “Data analysis”
28. Participant checking	Did participants provide feedback on the findings?	N/A
**Reporting**		
29. Quotations presented	Were participant quotations presented to illustrate the themes/findings? Was each quotation identified? e.g., participant number	Section “Results”
30. Data and findings consistent	Was there consistency between the data presented and the findings?	Section “Results”
31. Clarity of major themes	Were major themes clearly presented in the findings?	Section “Results”
32. Clarity of minor themes	Is there a description of diverse cases or a discussion of minor themes?	Section “Results”

**Fig. 1:**
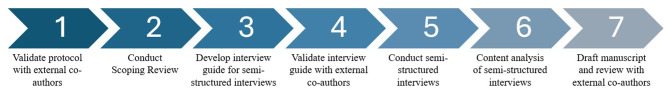
Study approach by steps

The study was conducted following an exploratory phase, given the scarcity of research on precision psychiatry in LATAM and the novelty of the topic. The exploratory phase included preliminary conversations with experts in the field to help inform the focus of this study and approach. A study protocol was developed and validated with five experts that act as co-authors in this study (AS, CLJ, EV, HC, JRB) through two rounds of feedback. Then, the study followed the seven-step process as described in Fig. 1.

A scoping review was carried out to inform the initial development of the interview guide. The scoping review focused on three overarching topics: (1) definitions of precision psychiatry, (2) benefits and challenges of precision psychiatry; (3) precision psychiatry and MDD. The methodology regarding search terms, outcomes to be extracted, and screening process was kept purposefully broad to allow the inclusion of studies with different designs, objectives, and methodologies, given the novelty of the topic. As drivers and barriers for precision psychiatry were not expected to be region-sensitive, studies from both LATAM and from a global perspective would also be applicable to LATAM. The research was conducted using a wide range of sources, including peer-reviewed literature, reports, governmental websites, factsheets, and policy documents. The findings of the scoping review were used to develop the guide for the semi-structured interviews, which was subsequently validated with the five experts included as co-authors.

### Participants and recruitment

A purposive sampling strategy for selecting study participants was carried out, to ensure that the participants had experience in the topic of precision psychiatry and could provide an informed and robust opinion on the topic, also aiming for diversity of participants from different countries and backgrounds.

Argentina, Brazil, Colombia, Mexico, Panama, and Peru were selected as the Latin American focus countries for expert recruitment, as they represent diverse and illustrative contexts within the region. These countries were chosen due to their regional relevance and variation in MDD prevalence and health policy approaches. Together, they reflect different socioeconomic settings [25], as well as geographic diversity across the region, thereby providing a heterogeneous backdrop for the study. While not fully representative of all LATAM countries, this selection captures a range of healthcare systems, epidemiological profiles, and policy environments, allowing the study to explore perspectives that are broadly informative for the region.

The strategic scan of experts consisted of web-based searches to identify stakeholders with academic merit, expertise, knowledge, and policy influence on the topic, thus who could provide valuable insight on the value drivers and barriers of precision psychiatry in LATAM. Three profiles were considered in the scan, with inclusion criteria tailored to capture the perspectives of key stakeholders most relevant to precision psychiatry implementation (Table 2). For the first two profiles, participants should be from one of the focus countries. For the third profile, participants from outside LATAM were also identified as the research field is still evolving and the literature on precision psychiatry by LATAM authors is still scarce. Furthermore, a global perspective could offer valuable insights from countries where the topic may be more advanced, highlighting implementation strategies and challenges that could inform LATAM-specific contexts, thereby enabling cross-country learning.

**Table 2:** Criteria for selection of participants

Profile	Inclusion Criteria
Healthcare providers with expertise in MDD	Experts with experience in the challenges faced by providers in the provision of diagnosis, treatment, and care to MDD patients, or renowned local psychiatrists specialized in MDD, preferably with linkages to relevant medical societies. Experts in this profile bring their clinical knowledge and understanding of care pathways to provide specific insights into how precision psychiatry can be applied to MDD.
Mental health policy influencers	Experts with general experience with mental health policies and policymaking processes in focus countries, preferably with experience influencing/advocating for precision medicine within mental health. Experts in this profile have brought their experience in the design/influence of policies, care pathways, protocols and other decision-making roles to provide insights on how the complex interplay between scientific advancements, regulatory frameworks, and societal needs may act as value drivers or barriers to the implementation of precision psychiatry.
Precision psychiatry experts	Professionals with a strong academic profile within precision psychiatry, according to publications in peer-reviewed journals, including individuals with considerable experience in precision psychiatry applied to MDD (whenever possible). Experts in this profile understand the intricacies of precision psychiatry and can provide a very realistic picture of the drivers and barriers for its implementation.

The first part of the strategic scan consisted of identifying a broad pool of potential participants who could meet the study criteria. A total of 85 potential participants were identified and categorized according to the three profiles presented in Table 2. Each potential participant was then assessed based on the context and volume of relevant academic publications, familiarity with the concept of precision psychiatry, and level of influence in policy or decision-making. Potential participants were classified as fitting one of the three profiles, but many of them also overlapped with other profiles.

A purposeful sample of 17 experts was initially identified as those that more strongly met the criteria outlined, also ensuring balanced representation of the three profiles and focus countries. This purposeful approach is appropriate when researchers need to obtain information from experts most likely to possess the knowledge and experience required to answer the research questions. Participants were contacted by email and 12 chose to participate in the study; of the five participants who did not take part, two declined the invitation, and three did not respond to follow-up emails. The strategic sample included experts from Argentina, Brazil, Canada, Colombia, Germany, Italy, Mexico, Panama, and Peru, and included psychiatrists, professors, senior managers, researchers, and members of relevant medical societies. After completing 12 interviews, no new themes related to the drivers, barriers, or perceived value of precision psychiatry emerged, and the research team therefore decided not to invite additional experts. The final sample size was adequate to meet the objectives of the study and ensured that interviews were information-rich and thematically focused [26, 27]. Table S1 in the Supplementary Material summarizes participant characteristics.

### Data collection

Data collection was conducted using a semi-structured interview guide with open-ended questions, which were the same for all participants. The guide included questions regarding local understanding and awareness of precision psychiatry, current local challenges in treating MDD, potential role of precision psychiatry in local MDD treatment, local drivers and barriers to adoption, and future implementation and local stakeholder involvement. Regarding the understanding of precision psychiatry, the presentation of a fixed or operationalized definition to participants was intentionally avoided during the interviews to allow them to articulate their own understanding of the concept without constraint. The full interview guide is presented in Table S2 of the Supplementary Material.

Interviews were conducted in April 2025 by two co-authors (MQ and JVR), either in English, Portuguese, and Spanish, according to the participants choice, with MQ and JVR fluent in the three languages.

The interviews were conducted virtually via MS Teams, where they were both recorded and transcribed through the platform’s built-in features. Each session lasted approximately 90 min. Transcriptions were entered into NVivo qualitative data analysis software (NVivo, version 15.1.2) in the original language of the interview. Analysis was conducted using verbatim transcription to preserve nuance. Thematic coding and development of themes were conducted in English by the two researchers fluent in the three languages to maintain consistency in interpretation. To present illustrative quotations and main findings in English, translation was carried out using a forward-backward method with Google Translate, and both researchers cross-checked translations to ensure accuracy of key concepts and preserve meaning.

### Data analysis

Data was analyzed through qualitative content analysis using a hybrid deductive-inductive approach, which was considered well-suited for exploring a complex and emerging topic such as precision psychiatry, the exploratory nature of the study, and the limited existing theoretical frameworks. The scoping review was first conducted to guide the development of the interview guide and ensure alignment with the study objectives, and the overarching themes were defined deductively based on these. Within these overarching themes, subthemes were derived inductively from participants’ responses, allowing new categories and nuances to emerge naturally without prior expectations. This hybrid approach ensured that the analysis remained grounded in the data, capturing the rich and context-specific perspectives of the experts, while maintaining a clear focus on the predefined study objectives.

After the transcriptions of interviews were introduced in NVivo, coding was conducted through iterative stages between May and August 2025: two authors (MQ and JVR) independently coded an initial subset of transcripts that led to a preliminary set of subthemes. The two researchers then discussed and conducted additional rounds of coding, repeatedly reviewing and updating the subthemes to incorporate new subthemes that emerged and refine the existing ones. Finally, the researchers held a discussion to ensure inter-coder reliability, confirm the organization of subthemes within the broader analytical themes, and resolve any discrepancies through consensus. Quotations have been selected to illustrate the findings, using NVivo.

The findings of the content analysis were validated with the five experts included as co-authors (AS, CLJ, EV, HC, JRB). Findings from the content analysis and scoping review were synthesized in a draft manuscript. To enhance interpretability, findings are presented aligned to domains of the Consolidated Framework for Implementation Research (CFIR): Innovation, Outer Setting, Inner Setting, Individuals, and Process [28]. Triangulation was conducted by integrating interview data with insights from the literature review to integrate multiple perspectives, and the manuscript was then reviewed, validated and finalized with the help of the external co-authors.

### Research team and reflexivity

The research team that collected, analyzed, and interpreted the data, and prepared the first draft of the manuscript consisted of MQ (MD, psychiatrist) and JVR (PhD, Public Health). Both are originally from LATAM, MQ currently living in a non-focus LATAM country and JVR in Europe. MQ brings clinical and health policy experience, while JVR contributes expertise in qualitative methodology. Their roles during interviews were limited to facilitating discussion and clarifying responses, which helped preserve participants’ perspectives. HF (BSc, Political Science) is based in the United States and contributed to study design and manuscript review, providing health policy expertise. Five co-authors (AS, CLJ, EV, HC, JRB), all psychiatrists with clinical and academic experience, reviewed and validated the study protocol and interview guide. AS and EV, based in Europe, provided additional conceptual methodological guidance on this emerging topic, while CLJ, HC, and JRB, based in focus countries, ensured contextual relevance for the LATAM landscape. None of the authors shared affiliations with participants or had relationships with them during the study, and analytical independence was ensured by assigning primary responsibility for data analysis exclusively to MQ and JVR, ensuring that findings remained grounded in participants’ perspectives rather than shaped by researchers’ assumptions.

### Ethical statement

This study received ethical approval from the Research Ethics Committee CAIMED, Colombia (N° CEIC-01440-25) in March 2025, and the study was performed according to ethical principles of the Declaration of Helsinki. All participants received written information and gave written consent to participate in the study and for publication of data before the interviews started. Participants consent to have their names included in the acknowledgments, and for their responses to be used in the study without direct attribution.

## Results

The results of this study are based on the data analysis of the 12 interviews with experts, in which we identified six major themes as follows: (1) Conceptualization and Understanding of Precision Psychiatry; (2) Challenges in Current Psychiatric Practice; (3) Value and Drivers of Precision Psychiatry; (4) Challenges and Barriers of Precision Psychiatry; (5) Current Status of Precision Psychiatry; (6) Recommendations. Within those, there were 35 sub-themes identified. The themes and sub-themes that emerged from the content analysis, as well as some key illustrative quotes from the interviews, are presented in Table 3. A more comprehensive set of interview excerpts is available in Table S3 of the Supplementary Material to provide additional context and depth.

**Table 3:** Summary of themes, subthemes, and findings

Theme	Subtheme	Definition	Quotes
1. Conceptualization and Understanding of Precision Psychiatry	Conceptualization of Precision Psychiatry	Experts have varied interpretations of precision psychiatry, encompassing group stratification, biomarker-based diagnosis, targeted treatments informed by genetics, lifestyle, and environment, the use of predictive algorithms and pharmacogenomics, and a patient-centered approach prioritizing well-being.	“Establishing a diagnosis or treatment based on specific biological characteristics or specific biomarkers.”
	Confusion around the concept of PP	Experts acknowledge confusion around the definition of precision psychiatry, including the interchangeable use of terms like “precision psychiatry,” “personalized psychiatry,” and “individualized psychiatry,” as well as the misinterpretation that it highly depends on artificial intelligence.	“Targeted treatment is more closely linked to personalized, individualized medicine, which is similar but not identical to precision medicine. Targeted means looking at the specific characteristics of an individual and then choosing a treatment that is good for that particular patient.”
	Familiarity of healthcare providers and patients	There is limited familiarity with precision psychiatry among healthcare providers, patients, and decision-makers, though younger professionals and those in academia may be more familiar.	“Precision psychiatry is mostly discussed in academic circles or among particular interest groups, and even within that small expert community, there are different understandings of what it actually means.”
	Main sources of information	Experts primarily rely on academic publications and professional events (e.g., congresses, workshops) as sources of information on precision psychiatry.	“Some conferences and roundtables attract a lot of attention, but the professionals attending these talks often leave them somewhat frustrated because the content of the presentations is very limited.”
2. Challenges in Current Psychiatric Practice	Heterogeneity of psychiatric conditions	The heterogeneity of depression complicates diagnostic and treatment decisions, as current systems often group patients with widely varying symptoms under the same label, failing to account for the complexity and subtypes of these conditions.	“We are not precise enough when diagnosing patients, and in practice, we study patients who are very different from one another, even though they carry the exact same diagnosis.”
	Reliability of diagnostic methods	Current psychiatric diagnostic systems have adequate inter-rated reliability and are effective for screening, but lack validity for nuanced diagnoses, with guidelines not aligning well with clinical practice, complicating accurate diagnosis.	“The reality is that our clinical evaluations still have some limitations; however, the use of diagnostic systems has an acceptable degree of reliability, which we rely on to establish our diagnoses.”
	Awareness, stigma, and health-seeking behaviour	Lack of awareness and stigma regarding mental health prevent many from seeking timely psychiatric care, contributing to delays in treatment.	“Stigma has decreased over the past decades, but the patient still resists seeing a psychiatrist. Strangely enough, they prefer to be treated by a non-specialist.”
	Availability and Knowledge of Healthcare providers	There is a shortage of psychiatrists, leaving many patients to face long wait times or receive care from non-specialists with limited expertise, hindering access to quality mental health services.	“The system does not have enough psychiatrists to handle all psychiatric cases. As a result, patients end up being treated by other professionals: non-specialists, general practitioners, neurologists.”
	Access to medicines	Access to psychiatric medications is limited by high costs, inconsistent availability, and restricted formularies, especially in public health systems where treatment options can be scarce and unreliable.	“There’s a lack of available treatment options, almost no non-pharmacological measures are offered, and pharmacological ones are very limited, often with significant side effects and safety concerns.”
	Impact on outcomes	Challenges in current psychiatric practice can lead to delayed diagnosis of depression, poor treatment adherence, frequent relapses, increased hospitalizations, and treatment-resistant cases. These issues negatively impact patients’ functioning, work performance, social relationships, and overall quality of life, while also increasing long-term healthcare costs and reducing human capital.	“Delays in starting treatment mean patients experience more depressive episodes and more hospitalizations due to untreated depression. These delays affect quality of life, the ability to function, return to work, and maintain family and social relationships, and many outcomes worsen over time.”
3. Value and Drivers of Precision Psychiatry	Support Clinical Decision-making	Precision psychiatry could lead to a more proactive and preventative model by supporting biologically precise diagnoses, grouping patients into more homogeneous subtypes, and tailoring treatments to patient needs.	“A biological individualization of these patients into more homogeneous biological subgroups will certainly enable more effective treatments with fewer side effects.”
	Clinical benefits	Precision psychiatry could enable early identification of patients, tailor treatments to individual needs, reduce exposure to ineffective therapies, thereby enhancing treatment efficacy, reducing side effects, accelerating recovery, and lowering the risk of long-term complications.	“The goal is to identify patients who are likely to benefit from these therapies earlier. Early identification and effective treatment would improve outcomes, reduce complications, and enhance efficacy while minimizing exposure to side effects.”
	Health-system and societal benefits	Precision psychiatry could enhance patients’ quality of life and functional outcomes, as well as optimize resource use, reduce healthcare costs, and improve societal productivity, benefiting individuals, the broader healthcare system, and society.	“Over time, from an economic and health outcome perspective, expenses should improve. A stable patient means fewer consultations, fewer hospitalizations, and increased productivity. So overall, the long-term impact would be positive.”
	Benefits of Precision Psychiatry for MDD	MDD cases that would benefit most from a precision approach are those with severe symptoms or complex presentations because these cases involve more objective symptoms with high variability in treatment response: sleep disturbances, anhedonia, cognitive symptoms, suicidal ideation, and psychotic features.	“There are some that are especially hard to treat, like anhedonia and cognitive symptoms. These would benefit most from precision approaches.”
	Receptiveness by healthcare providers and patients	Receptiveness among healthcare providers and patients will likely be high as approaches could align with current practices and would improve clinical efficiency and treatment outcomes. Resistance is expected to be temporary as the benefits of precision psychiatry become evident.	“I think that the main advantage is that these professionals are very interested and receptive to this kind of approach. In other words, if we have even minimal scientific evidence to guide professionals toward the best treatment for each patient, they are very open to it, and so are the patients.”
	Pathways to Establish Precision Psychiatry	Biomarkers and genetic markers show promise but have not been adequately established yet. Effective precision psychiatry requires integrating categorical diagnoses with complementary approaches, considering broader pathways and systems of biology. Factors like social, behavioral, environmental influences, and lifestyle data are also crucial for advancing precision psychiatry.	“It is obvious that biomarkers and genetic markers are important, but only if they result in a product that actually improves treatment and the patient’s quality of life. If that does not happen, they are of no use.”
4. Barriers and Challenges of Precision Psychiatry	Supporting Evidence	The adoption of precision psychiatry is hindered by the absence of supporting evidence and compelling data to demonstrate its effectiveness, insufficient clinical trials, underdeveloped classification systems, and inadequate economic studies to confirm its benefits.	“This is a very important issue. On the one hand, we want to educate policymakers and stakeholders because, as clinicians, we believe in the concept of precision psychiatry. But on the other hand, we lack the evidence.” “We do not have proof that precision psychiatry actually works in practice.”
	Costs	The high costs of precision psychiatry, including development, implementation, and treatment, limit accessibility, with many patients unable to afford it and no payer to cover the costs.	“Ultimately, if patients are expected to pay out of pocket, many simply will not be able to. Precision medicine must become financially accessible to the end user, otherwise, it cannot be widely implemented, especially without a payer system in place. These treatments will likely be costly at first due to the complex integration of translational medicine, clinical care, and engineering.”
	Investments in Health	Mental health is rarely a government priority, and investment in mental health is minimal, with limited budgets for health and research.	“The situation is particularly difficult because there is not currently a substantial budget for health and research. Much of the work is carried out by research teams who secure limited funding, which makes advancing precision psychiatry quite difficult.”
	Regulatory Landscape	Regulatory processes for drug approval and incorporation are characterized by slow timelines, a focus on immediate costs rather than long-term impacts, difficulties conducting pharmacoeconomic studies, bureaucratic hurdles, difficulties in the assessment processes, and limited familiarity with precision psychiatry concepts.	“The public system looks directly at the cost of treatment. It has a great deal of difficulty conducting pharmacoeconomic studies. They only consider the immediate and direct costs. They do not consider the long-term impact this might have.”
	Mental Health Prioritization	Public health policies frequently lack technical expertise and are often driven by outdated or non-local guidelines rather than scientific evidence. Although mental health has gained more attention, it remains a low priority, with major gaps in effective public health strategies.	“In terms of public health strategies, we have very few targeted strategies for major depression treatment, almost nothing focused on this population.”
	Knowledge Among Health Care Providers	Many professionals face a lack of training and access to quality information, including in areas such as genomic analysis, especially among those who are not researchers.	“The other major challenge is the lack of training and lack of access to quality information for the vast majority of specialists, who are not researchers and are not based in universities. Research is not part of their daily routine.”
	Least Receptive Cases of MDD	Precision psychiatry would be less effective for symptoms tied to personality traits, social circumstances, and non-biological factors that show low variability in drug response and are difficult to change.	“Because social characteristics are also very important in precision psychiatry, and I think we need to include them. But on the other hand, they are hard to change. So social circumstances are difficult to modify, but I think it would be a mistake to exclude them.”
	Ethical considerations	Ethical issues include exposure to contrast materials or radioligands, the depersonalization of psychiatry, risk of errors in AI algorithms, and concerns about privacy and data misuse.	“There’s also everything related to privacy, because behind all this, even if it sounds very nice, our data will be used: for what? That hasn’t been addressed yet.”
5. Current and Future Status of Precision Psychiatry	Existing Experiences and Initiatives	There are few initiatives in precision psychiatry; most are limited to research or isolated efforts by specific institutions. Government-led programs are largely absent. Some experts mentioned having experienced practices they considered precision psychiatry, including tailoring treatments based on patient history and genetics, using pharmacogenomic testing, advanced neuroimaging, and innovative therapies like neuromodulation.	“Yes, we have off-label treatments today. For example, based on some studies and empirically, if a patient has treatment-resistant symptoms and hypercortisolemia, using a cortisol synthesis inhibitor tends to improve the patient’s response.”
	Time for Realization	Precision psychiatry is still far from widespread implementation, needing more investment in patient stratification and reliable biomarkers. Progress will take time, requiring advances in technology, treatment trials, and the use of tools like AI. Significant impact is unlikely soon, though some markers may become useful in the near future.	“At the moment, I am not aware of any very strong biomarker that is widely usable or accepted.”
6. Recommendations	Generate Evidence	Generating robust scientific evidence is essential to demonstrating the value of precision psychiatry. By combining pharmacoeconomic studies with quality-of-life analyses, it is possible to highlight both its broader economic and societal benefits, driving its adoption.	“Critical factors include first providing evidence. We must generate and demonstrate that precision psychiatry works and offers benefits compared to conventional treatment. This requires a major effort to generate that evidence.”
	Expand Awareness and Training in Precision Psychiatry	Training programs are crucial for equipping healthcare providers, especially psychiatrists, with the knowledge and tools needed to successfully implement precision psychiatry. This can be achieved through continuing education programs for specialists, webinars, symposiums, outreach campaigns, social media platforms, and incorporation of precision psychiatry into medical curricula.	“Critical factors for successful implementation include knowledge, as education and training of local healthcare providers are crucial; they are the gatekeepers for precision psychiatry’s success and commercialization.”
	Improve Health Technology Assessment Processes	Regulatory bodies should integrate health technology assessments that combine clinical and economic evidence to highlight cost-effectiveness and societal benefits. Clear recognition of these advantages by policymakers and regulators is key to facilitating adoption.	“Basically, what they need to know is how these new techniques can affect their costs. Costs not only in terms of money, but also in terms of time that doctors have to spend with a patient before the patient feels well; the time the patient has to spend in the hospital before they improve; the time the patient must stay off work, which is also a cost to society.”
	Increase Funding for Mental Health and Research	Increased funding is essential to gather evidence, train psychiatrists, expand practical knowledge, and implement precision psychiatry effectively.	“We also need to be honest and admit that we do not yet know the full potential benefits of precision psychiatry. We assume there are some advantages, but scientifically, this is still uncertain. We definitely need more funding programs for research in precision psychiatry.”
	Strengthen Mental Health Policies	Strengthening mental health policies and fostering political will is essential to overcome the challenges posed by outdated decision-making, limited expertise in public health, and current gaps in current psychiatric practice.	“We need changes at the level of public mental health policies. I believe the critical factor for implementation is to have a strong, organized proposal or team and political will to make it happen.”
	Engage Stakeholders	Effective implementation of precision psychiatry requires broad interdisciplinary stakeholder engagement, including researchers, healthcare providers, industry partners, policymakers, regulatory bodies, patient organizations, families, and medical associations. Close communication and collaboration among these diverse groups are essential.	“I think we need to not only look at researchers, but also include industry partners, policymakers, and stakeholders such as patient organizations. So, it must be a broad interdisciplinary approach. Bringing them together is a critical factor for generating evidence and planning for implementation, fostering joint understanding. This requires closer communication between industry, researchers in precision psychiatry, and policymakers.”
	Enhance Healthcare Service Provision	Addressing challenges in healthcare service provision, such as improving specialist availability, establishing early diagnosis pathways, and ensuring well-equipped hospitals, is essential to enhance care quality, expand access, and enable the effective implementation of precision psychiatry.	“We may need a different approach, where not everyone with MDD first goes through primary care, then secondary, and then tertiary care. We need to build something that differentiates cases much earlier.”
	Improve Integration and Management of Healthcare Data Systems	Improving the integration and management of healthcare data systems is crucial for achieving interoperability of electronic health records, leveraging AI tools, aligning medical records across systems, and enabling effective patient monitoring, key components for advancing precision psychiatry.	“The information I can access about a patient is only available to the patient and me in my medical record. But if the patient sees another colleague, that information probably will not be accessible or useful for improving care.” “I think there needs to be alignment with local medical records, digital records, and electronic records. We are talking about different healthcare systems, so to demonstrate impact, we’ll need large, integrated population data. That will be a challenge in some jurisdictions.”
	Leverage International Experience	Building upon the knowledge and best practices gained from international experiences in precision psychiatry, analyzing advancements from other countries, and adapting them to the national context.	“I would suggest presenting external experiences from countries that have already made progress in precision medicine, what they have achieved, and how we could apply that to our population. Of course, this should consider the genetic characteristics of our population, which are very diverse. We would need to apply or adapt their advancements accordingly. “

### Analysis of major themes

Findings are presented in alignment with CFIR domains [28]: (1) Intervention Characteristics: attributes of precision psychiatry itself such as perceived benefits, complexity, evidence base, and cost; (2) Outer Setting: external influences including policy environment, regulation, funding structures, and societal factors; (3) Inner Setting: organizational and health system context, including infrastructure, culture, and readiness for implementation; (4) Characteristics of Individuals: knowledge, beliefs, attitudes, and receptiveness; and (5) Process: planning, stakeholder engagement, execution strategies, and future implementation efforts.

#### Precision psychiatry characteristics

Experts had varied interpretations of the concept of precision psychiatry. Some of the ideas mentioned by experts include tailoring care using biological markers and individual factors, to treat the individual from their own point of view, as well as data-driven approaches that integrate complex datasets to enable patient subtyping and targeted treatment. They also reported confusion around the concept, including overlaps between “precision, personalized, and individualized medicine”, and the common assumption that it relies heavily on artificial intelligence.

Experts identified several potential benefits of Precision Psychiatry, with its most frequently mentioned value being its ability to support and enhance clinical decision-making. Experts agree that precision psychiatry, through biological individualization, symptom clustering, and identification of biomarkers, has the potential to transform mental health care. Tailoring treatments to more biologically and symptomatically defined subgroups, could lead to improved treatment effectiveness, reduced side effects, shorter diagnostic and therapeutic timelines, improved follow-up, and enhanced patient adherence and outcomes. Experts highlight that shifting from generalized, trial-and-error approaches to data-driven, individualized care in MDD could significantly improve outcomes. Early identification based on symptom patterns, severity, and risk factors, supported by medical records and big data, would enhance the risk-benefit ratio, ultimately leading to better long-term recovery and quality of life. Experts agree that precision psychiatry would enable more proactive, preventive care by targeting dominant symptoms. This potential impact on clinical decision-making would support psychiatrists’ confidence in their practice, and experts believe that both healthcare professionals and patients would be receptive to precision approaches.

Experts mentioned that the potential value of precision psychiatry in clinical practice would also positively impact health systems and society as a whole: by improving clinical outcomes and reducing ineffective care, it enables a more efficient use of healthcare resources. This not only lowers long-term costs related to consultations, hospitalizations, and disability, but also enhances patients’ ability to function in daily life, leading to broader social and economic benefits such as increased productivity.

Regarding MDD, experts highlighted that a precision approach could be especially beneficial for symptom domains such as sleep disturbances, cognitive impairments, and anhedonia, which are often harder to treat and show high variability in treatment response. These symptoms are more objective and less heterogeneous across patients, making them suitable symptoms for targeted interventions. Suicide risk, treatment-resistant depression, and severe or recurrent cases were also identified as key priorities due to their complexity and potential for long-term consequences.

Despite its perceived potential to improve health outcomes, experts also highlighted intrinsic characteristics of precision psychiatry that may hinder its implementation and pose significant challenges to its adoption, with the absence of supporting evidence and compelling data to demonstrate its effectiveness and cost-efficiency being the most cited barrier. Experts emphasized that the field remains at an early stage, with limited rigorous research and no clear strategies for integration into health systems. Progress is constrained by insufficient supporting evidence, a lack of robust clinical trials, underdeveloped classification frameworks, and limited economic evaluations to demonstrate cost-effectiveness. Moreover, certain aspects of MDD, such as psychosocial stressors, present additional challenges regarding generating evidence and being receptive to precision psychiatry, as these factors are less directly linked to biological mechanisms.

The high costs associated with developing, implementing, and accessing precision psychiatry, at least in its first stages of implementation, were also identified as significant barriers to its accessibility. Although the technologies themselves are not always invasive, building models, conducting genetic tests, and integrating clinical and computational expertise require substantial investment.

Finally, experts also highlighted ethical concerns related to precision psychiatry. These include the high costs and potential risks associated with advanced technologies, which may expose patients to contrast agents or radioligands; the risk of depersonalization in psychiatric care and the possibility of diagnostic errors from AI models; and privacy and data protection issues.

#### Outer setting

This section outlines the external contextual factors that, according to experts, influence the implementation of precision psychiatry. There are challenges in the current psychiatric practice that can shape the implementation of precision psychiatry: experts highlight that current diagnostic systems oversimplify depression, failing to account for its diverse symptom profiles and subtypes, which can lead to imprecise diagnoses and treatments. In fact, the heterogeneity of depression is identified by experts as one of the main challenges in managing the disease, with one expert highlighting that this is the main reason why psychiatry lags behind other fields, such as oncology, in advancing precision medicine. Experts expressed that despite the reliability of current psychiatric diagnostic methods (i.e., agreement between professionals is often high), the validity of these diagnoses remains very low. They also noted that although manuals like the Diagnostic and Statistical Manual of Mental Disorders (DSM) and International Classification of Diseases (ICD) have standardized language and criteria, they tend to oversimplify complex conditions and overlook important clinical nuances that psychiatrists observe in their clinical practice.

Experts identified other challenges in diagnosing, treating, and managing follow-up for MDD, including patient-related factors such as limited awareness, stigma, and low health-seeking behavior; provider-related factors such as insufficient training and knowledge; and systemic issues like limited access to medications within the healthcare system. Experts emphasized that stigma remains a significant barrier to mental health care, particularly for depression. Despite some progress, many patients still avoid psychiatric help, lack awareness about depression, or delay treatment until symptoms become severe. One expert also pointed to structural stigma, such as limited insurance coverage for psychiatric care, while another expert discussed stigma between healthcare providers, where even psychologists may hesitate to refer patients to psychiatrists due to their own biases.

Experts highlighted that access to effective pharmacological treatment for depression is severely limited. Medications are often expensive and frequently not fully covered by the health system. Some experts highlighted that the list of medications included in national health plans is often limited and outdated, with some medications associated with significant side effects and safety concerns, which can lead patients to abandon the treatment, while newer medications face bureaucratic delays for approval. In addition, medications are not always consistently available, leading to a lack of continuity in care. One expert mentioned that non-pharmacological options are also scarce, leaving patients with few safe and effective treatment choices.

Many patients in LATAM will not be able to afford innovative treatments or diagnostic tools, and public health systems often restrict access due to budget constraints. Experts expressed that without a payer covering these expenses, out-of-pocket costs become a major barrier, making widespread adoption of precision psychiatry unlikely unless it becomes financially viable for both patients and health systems. This is concerning given that, as experts reported, the focus countries face significant budget constraints for health and research, limited investment in mental health, and poor management of existing resources.

Mental health was reported as a low priority in LATAM, exemplified by a lack of investment and strategy in mental health, and there are still very few targeted public health strategies, particularly for major depression, despite growing interest in recent years. Structural stigma persists, as policies and public attitudes continue to marginalize people with mental health conditions and view them as a burden. Experts highlight that public policy challenges also stem from policymakers who lack up-to-date, practical experience in mental health, have a short-term focus, and may base decisions on ideology or political agendas rather than solid scientific evidence.

Experts describe regulatory processes in Latin America as slow and bureaucratic, with significant delays in approving new treatments. These systems tend to focus narrowly on immediate and direct costs, often neglecting long-term impacts and struggling with pharmacoeconomic evaluations. Strict protocols and unfamiliarity with concepts like precision psychiatry further complicate the introduction of innovative treatments, particularly within public health systems.

Regulatory processes in Latin America are described as slow and bureaucratic, with significant delays in approving new treatments. Some experts reported that regulators’ systems tend to focus narrowly on immediate and direct costs, often neglecting long-term impacts and struggling with pharmacoeconomic evaluations. Experts also note that regulators often rely on outdated treatment guidelines based on conventional psychiatry, which hinders the adoption of innovative approaches like precision psychiatry. Although it was not found clear evidence that psychiatry treatment guidelines are outdated in Latin America, it is essential to ensure that they are regularly updated given scientific and technological advances transforming the field, including developments in precision psychiatry.

#### Inner setting

This section examines organizational and health system factors that will affect the readiness and capacity to implement precision psychiatry according to experts. Experts expressed that in the absence of concrete data showing improved outcomes and cost savings, policymakers and organizational decision-makers within the inner health system remain skeptical and tend to view new technologies as added expenses rather than worthwhile investments, and regulatory approval and public reimbursement will require compelling evidence. This is a significant challenge for precision psychiatry, as one of the main issues raised by experts is that most of the discussion on the benefits of precision psychiatry is hypothetical, with not enough robust evidence to support it.

Another factor in the inner domain that may impact the implementation of precision psychiatry is the available workforce. The shortage of psychiatrists was reported as a major barrier to mental health care in the focus countries, which can lead to long wait times, often several months from initial primary care to psychiatric appointment, creating a significant barrier to accessing specialized mental health care. Due to psychiatrist shortages, many patients are treated by general practitioners or psychologists, who often lack the training or prescribing authority to manage moderate to severe cases effectively. One expert pointed out that even among psychiatrists, many lack formal residency training, leading to limited expertise or experience when conducting clinical diagnostic evaluations. Regarding precision psychiatry itself, experts noted that limited training and understanding of precision psychiatry, including genomics, remain challenges for psychiatrists, although this was mentioned less often than other barriers, likely due to the field’s novelty and limited real world use.

#### Characteristics of individuals

This section presents experts’ views on how individual knowledge, beliefs, and attitudes may influence the adoption of precision psychiatry, a topic that received limited attention. While healthcare providers are generally familiar with the term, many lack understanding of its specific methods or tools, although the concept is increasingly recognized among newer generations of psychiatrists. Patients tend to have minimal knowledge, and decision-makers usually lack the technical expertise needed to engage with the topic. Discussions are mostly limited to academic circles, where even among specialists, definitions vary. Experts cited conferences, international publications (mainly English-language literature), and pharmaceutical company summits as their main sources of information.

#### Process

This section outlines the perceived processes, pathways and strategies involved in implementing precision psychiatry. Although experts recognize the potential benefits of precision psychiatry, differing views emerged when discussing the pathways to its implementation. One expert noted that without clear biomarkers or genetic evidence, precision psychiatry has not yet proven its clinical or economic value. Another emphasized that biomarkers are only meaningful if they lead to treatments that improve outcomes and quality of life. In addition, biomarkers and genetic data are currently tied to existing diagnostic classifications, which present limitations on the need to unify brain measures with extracerebral factors or capture heterogeneity in individual trajectories [29].

Some experts highlighted the need to look beyond only biomarkers and genetic data when advancing precision psychiatry. They emphasized the importance of examining broader biological systems and neurobiological pathways, such as those related to inflammation or neurotrophic factors, rather than focusing solely on individual genes or biomarkers. Understanding dysregulated pathways can inform treatment decisions even in the absence of specific genetic tests. In clinical practice, experts also emphasized a holistic clinical approach that includes psychosocial, behavioral, and environmental factors, noting that even with biomarkers, understanding a patient’s context is essential for effective treatment.

A few experts mentioned digital tools and innovations that can support data collection and monitoring, including patient-generated mood logs, digital phenotyping, and data related to lifestyle, physical activity, diet, sleep, and family history, which can be facilitated through mobile applications and wearable devices, some of which are already being used in practice according to some experts. They also emphasized the value of incorporating neurophysiology, neuroimaging, clinimetrics, and data from electronic health records and digital lifestyle tracking in precision algorithms.

Experts from LATAM identified certain activities in their current practice or referenced initiatives they consider aligned with a precision approach to psychiatry, including tailoring treatments based on patient history and environmental factors, using pharmacogenetic and pharmacoeconomic tests, and applying cognitive behavioral therapy for specific symptoms like insomnia. Some mentioned off-label treatments guided by clinical patterns, such as using cortisol synthesis inhibitors in resistant cases. Access to advanced tools like neuroimaging, neuromodulation, virtual reality, and software that analyzes facial expressions as an auxiliary diagnostic tool was also mentioned. However, most of these practices are limited to research or isolated efforts by specific institutions, with experts noting a lack of validated biomarkers and minimal to no government-led or public initiatives.

Experts mentioned varied advancements needed to ensure that precision psychiatry advances in the region, which include a shift toward stratified research, focusing on specific subtypes of mental disorders rather than broad diagnostic categories, studies targeting more homogeneous patient groups, development of reliable and accessible diagnostic tools, randomized clinical trials to identify treatment-specific markers, integration of genetic and family history data, and to improve data collection during clinical interviews incorporating technologies like artificial intelligence and machine learning. However, experts noted that progress is slow, estimating that it may take five to ten years to become a practical reality, citing limited validated biomarkers, ethical concerns around data use, and the need for further development and implementation of innovative tools.

#### Recommendations

Based on the insights provided by the experts interviewed and the main challenges and barriers identified, nine key recommendations were identified to drive the advancement of Precision Psychiatry in Latin America, as presented in Table 4.

**Table 4:** Recommendations for the implementation of precision psychiatry in Latin America

Barriers and challenges	Recommendation	Importance of recommendation
Supporting evidence: *Lack of supporting evidence and compelling data to demonstrate its effectiveness and cost-effectiveness*	Generate Evidence	It is essential to generate robust evidence of clinical effectiveness or precision psychiatry and also of its socioeconomic impact. Policymakers and stakeholders are more likely to support new approaches when presented with clear data demonstrating improvements in outcomes alongside reductions in costs, hospitalizations, and resource use. There is a need to generate evidence that is specific to Latin America to ensure relevance and applicability. These real-world studies and pharmacoeconomic research should measure both individual benefits and broader societal gains.
Awareness, stigma, and health-seeking behavior: *Lack of awareness regarding mental health.* Knowledge of healthcare providers: *Shortage of psychiatrists; lack of training and access to quality information*	Expand Awareness and Training in Precision Psychiatry	Training healthcare providers, especially psychiatrists, with the necessary knowledge and tools is crucial for the successful implementation of precision psychiatry. Many psychiatrists currently lack understanding of key areas like multi-omics, neurophysiology, neuroimaging, and evidence-based medicine, making ongoing training essential. Promoting training in precision psychiatry can be achieved through comprehensive, ongoing continuing education programs for all specialists; integration into medical, psychology, and public health curricula; active involvement of professional associations; and leadership by specialized mental health centers.
Supporting evidence: *Absence of compelling data to demonstrate its cost-effectiveness and benefits* Costs: *High costs of precision psychiatry* Regulatory landscape: *Unfavorable regulatory processes*	Improve Health Technology Assessment Processes	A comprehensive health technology assessment should integrate both clinical and economic evidence, highlighting not only direct healthcare costs but also indirect costs related to patient productivity and societal impact. Demonstrating cost-effectiveness through pharmacoeconomic studies and quality-of-life comparisons will help convince policymakers and regulators of the value of precision psychiatry. Regulatory bodies may face challenges in adapting to novel study designs related to precision psychiatry, making early dialogue between pharmaceutical companies, psychiatric societies, and regulators crucial to align methodologies and expectations.
Costs: *High costs of precision psychiatry*,* including development*,* implementation*,* and treatment* Investments in health: *Limited budgets in health and research*	Increase Funding for Mental Health and Research	Increased funding is essential to the effective implementation of precision psychiatry, as it enables the comprehensive gathering of evidence on both its clinical efficacy and socioeconomic benefits. Investing in research and education is crucial to advancing the scientific understanding of precision psychiatry and translating these innovations into practice.
Mental health prioritization: *Low prioritization of mental health; lack of technical expertise*	Strengthen Mental Health Policies	It is essential to establish targeted policies that actively promote the implementation and advancement of precision psychiatry. Key priorities should include expanding government-led educational programs for psychiatrists and other healthcare providers, securing dedicated funding, supporting interoperable electronic health records, and continuously improving the provision of mental health care.
Awareness, stigma, and health-seeking behavior; knowledge of healthcare providers; supporting evidence; costs; investments in health; regulatory landscape; mental health prioritization	Engage Stakeholders	Effectively advancing precision psychiatry requires the involvement of a broad range of stakeholders across sectors. Key actors include researchers, universities, scientific societies, industry, the private sector, policymakers, regulatory bodies, and patient organizations. Collaboration between these key groups is essential to build a shared understanding of psychiatric disorders and to develop coordinated strategies for system improvement, ensuring the successful implementation of precision psychiatry.
Availability and Knowledge of Healthcare providers: *Shortage of psychiatrists* Access to medicines: *Limited access to psychiatric medications* Reliability of diagnostic methods: *Underdeveloped classification systems; low diagnosis validity.* Awareness, stigma, and health-seeking behavior: *Stigma regarding mental health*	Enhance Healthcare Service Provision	Addressing challenges in healthcare service provision requires expanding the system’s capacity to serve patients who need specialized care, both by increasing the availability of medications and ensuring access to trained mental health professionals. A more efficient model is needed to identify and differentiate complex cases like major depressive disorder earlier in the care pathway. Reducing stigma and strengthening psychiatry’s role as a recognized medical specialty is essential. Infrastructure improvements, such as equipping health facilities and expanding teleconsultations, are also critical.
Supporting evidence: *Need for comprehensive patient data* Ethical considerations: *Concerns about privacy and data misuse*	Improve Integration and Management of Healthcare Data Systems	Improving the integration and management of healthcare data systems is essential for advancing precision psychiatry. There needs to be better interoperability of medical records, as current systems often prevent patient information from being shared across providers. Establishing Precision Medicine Departments in public hospitals could help transform routine clinical data into valuable insights. Artificial intelligence tools offer promising opportunities to enhance data analysis.
Existing Experiences and Initiatives: *Limited local initiatives in precision psychiatry*	Leverage International Experience	Leveraging international experience is essential, as some countries are more advanced in precision psychiatry. Their experience can guide local adaptation, considering Latin America’s diverse genetic makeup and current limitations in tools and treatments.

## Discussion

Precision psychiatry is an emerging area of research that has received limited attention in Latin America. In this context, the present qualitative study aims to explore the perceptions of healthcare professionals regarding the value drivers and barriers to the adoption of precision psychiatry in the region. Importantly, the findings should be interpreted as reflecting the informed perceptions, experiences, and expectations of the participating experts rather than constituting empirical evidence of effectiveness, cost-effectiveness, or real-world impact.

Experts broadly agree that precision psychiatry could improve decision making and support a more proactive model of care by enabling precise diagnoses and tailored treatments. This may enhance outcomes, reduce side effects and complications, improve quality of life and functioning, and optimize healthcare resources and costs. Experts’ views align with existing reviews and theoretical papers, which suggest that precision psychiatry can enhance clinical management by integrating information from clinical assessments and system biomarkers to support decision-making [19, 30].

However, some divergence emerged among experts regarding the fundamental definition of precision psychiatry, as they offered varied interpretations and identified different experiences and initiatives they consider fitting the scope of precision psychiatry. Currently, there is no consensus on the definition of precision psychiatry, which can lead to diverse interpretations within the medical and research communities. The lack of a common definition can render it difficult to assess the implementation of precision initiatives and their benefits, hindering the advancement of this novel field. Some papers discuss that applying a precision approach to psychiatry means individuals being reclassified into different diagnoses and endophenotypes, which allows the provision of tailored interventions according to diagnostic subgroups, leading to better outcomes [19, 21, 22]. Nonetheless, a cohesive definition of precision psychiatry would facilitate the development of standardized protocols, enhance cross-disciplinary collaboration, and ultimately contribute to disseminating and adopting it into psychiatric clinical practice.

As experts noted, a foundational challenge for precision psychiatry is the difference in the nature of mental health disorders when compared to other diseases such as cancer and cardiovascular diseases: there is substantial symptom overlap and variability of mental disorders, with diagnosis relying on subjective assessment methods. Despite these challenges, new mechanisms of action have been recently developed for the targeted treatment of MDD, such as seltorexant (orexin pathway) [31], aticaprant (k-opioid receptor pathway) [32], daridorexant hydrochloride (dual orexin receptor antagonist) [33], ropanicant (nicotinic acetylcholine α4β2 receptor antagonist) [34], onfasprodil (negative allosteric modulator) [35], and navacaprant (kappa opioid receptor antagonist) [36]. The development of such medications hints that, despite some recent failures and the inherent complexity and diagnostic challenges in psychiatry, it is possible to identify and target specific biological mechanisms underlying mental disorders. Moreover, precision psychiatry approaches can also be applied beyond psychopharmacology, fostering neuromodulation and even psychotherapy [37].

In addition to the inherent heterogeneity of psychiatric disorders, experts identified several challenges in the outer and inner settings that may pose even greater barriers to the implementation of precision psychiatry, including low awareness, stigma, limited health-seeking behavior, insufficient availability of healthcare providers, gaps in their knowledge, and restricted access to medications. These represent important barriers to the adoption of precision psychiatry in LATAM, as some stakeholders could argue that public health policies in the region still struggle to address basic healthcare needs, leaving limited capacity for integrating advanced approaches. Therefore, addressing these issues is essential not only for the successful adoption of precision psychiatry but also for improving current mental healthcare systems more broadly.

Experts emphasized that, although precision psychiatry holds significant promise, the field is not yet sufficiently developed for widespread adoption. Besides the existing challenges faced in the current practice, there is still limited evidence of the benefits a precision approach to psychiatry, and limited evidence on cost-benefits from existing studies [38–40]. The potentially high costs of large-scale genetic testing and the elevated pricing of precision treatments raise concerns about the capacity of health systems to finance precision psychiatry. Experts discussed that it is then crucial to generate robust evidence not only demonstrating its positive impact on health outcomes but also clearly linking its benefits to resource savings and cost-effectiveness. Experts also emphasized the pressing need for clinical trials that randomize patients to different treatments to identify reliable biomarkers and demonstrate the effectiveness of precision psychiatry. The literature on the topic also indicates that the accelerated progress of precision psychiatry will require rapidly reactive regulatory frameworks, capable of successfully balancing priorities, ensuring patient safety while, at the same time, recognizing and incentivizing innovation [41–43].

Some initiatives have been launched in LATAM with the goal of advancing precision medicine in general, acknowledging the potential benefits it might bring to patients, healthcare providers, health systems, and drug developers. The Latin American Genomics Consortium was established in 2019 with the goal of exploring the genomic landscape of psychiatric disorders through collaborative efforts and engagement to increase sample size, expand recruiting and genotyping, investigate genetic methods development, and provide specialized training in psychiatric genomics research for investigators [44]. Efforts of similar nature can be found in Argentina, Brazil, and Colombia, where projects to map the genomes of the population are currently being implemented [45–47]. There is, however, still a scarcity of evidence of noteworthy results, highlighting the importance of further exploration to enrich a discussion that can inform the adoption of a precision approach in the field in LATAM.

In addition to the limited evidence supporting the adoption of precision psychiatry, experts identified several other barriers in LATAM, including high costs, low investment in healthcare, an unfavorable regulatory environment, limited prioritization of mental health, and inadequate knowledge among healthcare providers. Structural and system-level constraints further impact the feasibility of implementation, including limited funding for care, workforce shortages, fragmented and poorly coordinated health systems that hinder integration across providers and levels of care, and unequal access to services, which can exacerbate existing disparities in mental health care across populations and regions. These challenges highlight the need to bridge disciplinary and geographic boundaries and to mobilize resources to promote more equitable brain health outcomes across the region [48]. The low public and private investment in research and development in LATAM challenges the possibility of resolving the genomic representation gap and the development of innovative precision treatments. This gap is further intensified by the unique profile of social, environmental, and health-related risk factors in Latin America that influence mental health outcomes [49].

In addition, the limited availability of financial resources, medical specialists, and researchers, especially for training in the field of precision medicine and precision psychiatry, further challenges its adoption. In fact, evidence indicates poor perceived competence in and knowledge of genetic testing and pharmacogenomics among psychiatrists, posing a risk of low compliance towards adopting new tools [19, 38, 50].

When compared with evidence from Europe and North America, some of the barriers identified in Latin America appear to reflect broader global challenges in implementing precision approaches in psychiatry: a systematic review conducted primarily in high income countries highlighted barriers such as poor model accuracy and clinical utility, cost and time investments, limited professional competence in precision psychiatry, and potential psychological or economic harms [51]. These findings align with the uncertainty surrounding clinical benefit, financial constraints, and gaps in provider preparedness observed in the Latin American context. However, they also suggest that some challenges may be perceived as more significant in LATAM, or that additional barriers may exist and should be considered when planning implementation. Together, this comparison indicates that although the adoption of precision psychiatry faces common global obstacles, region specific structural and contextual factors must also be considered when designing implementation strategies.

There is an increasing need for strategies that support more accurate and timely diagnosis and treatment of mental health conditions, including MDD. As highlighted by experts, precision psychiatry has the potential to incorporate biological and environmental data to enhance treatments and complement clinical judgement when prescribing antidepressants, as well as contributing to therapeutic drug monitoring by integrating genetic testing and clinical assessment, with the ultimate goal of optimizing care [11, 13, 15]. The impact of MDD in LATAM and the challenges of current care models highlight the need to explore innovative approaches.

The literature indicates that the implementation of precision psychiatry would require an integrative approach of information from different domains (e.g., genetic, neurobiological markers, behavioral, and environmental), leading to more targeted and effective diagnosis and treatment approaches. The discovery of biomarkers through a comprehensive study of various components or aspects of a biological system is needed to lead to hypothesis-free and data-driven approaches in the neurobiology of psychiatric disorders [19, 21]. Such biomarkers must be combined with physiological recording and brain imaging for a more comprehensive understanding of neurological conditions beyond genetic correlations [19, 21, 52] and environmental exposure, behavioral, and self-reported data to ensure that models are person-centered and context-sensitive [19, 53, 54]. Addressing the complexity of interactions between environmental and health-related factors across the life course is therefore essential [55]. Furthermore, clinical presentation and the individual’s self-understanding and illness narratives must also be taken into consideration as they influence symptom interpretation and treatment response [53]. These various types of data must be appropriately collected, managed, integrated, and analyzed, requiring health workforce training and the incorporation of advances in computing, artificial intelligence, and machine learning [53, 56].

Therefore, implementation of precision psychiatry in clinical practice would require a synergy between different stakeholders, as the experts noted. Stakeholders would play diverse roles, including conducting and supporting scientific research, generating evidence, enhancing healthcare professionals’ knowledge, creating and improving data collection and processing tools, developing therapies, and setting strategic priorities and regulatory frameworks [19, 21, 50]. Together, these efforts aim to ensure that innovative technologies reach the patients who would benefit most, ultimately contributing to improved health outcomes and potentially reducing both direct healthcare costs and productivity losses.

### Strengths and limitations

This study’s strengths include the use of a hybrid content analysis approach, which allowed subthemes to emerge organically from the interview data, while aligned to the objectives of the study. Rigor was ensured through systematic coding, with inter-coder reliability strengthened through discussion and consensus-building. Additionally, member checking was employed to validate that the identified themes accurately represented participants’ perspectives. There are limitations in our study, particularly in participant selection, that require consideration when interpreting the results: the sample, selected through convenience sampling, is not representative of the broader psychiatric community. Participants were chosen for their demonstrated knowledge of precision psychiatry, as limited expertise would have constrained the depth and relevance of the discussions. However, selecting participants on the basis of recognized expertise may also have introduced a pro-innovation bias, as individuals actively engaged in or supportive of precision psychiatry may hold more optimistic and favorable views toward its development and implementation than the wider psychiatric community as well as from other medical specialties, potentially placing less emphasis on structural or conceptual limitations. The selection of a sample of elite experts on the topic may have privileged specialized viewpoints, potentially narrowing the range of perspectives captured. As is common in qualitative research, the sampling approach limited the diversity of perspectives, and the findings may not be fully generalizable to the wider population of psychiatric professionals.

Although some studies report that a sample size of 12 may be adequate to address a research question [57, 58], we cannot ensure that data saturation was fully achieved given the novelty of precision psychiatry, the exploratory nature of the study, the purposive sample strategy, the broad scope of the interview questions, and the potential for diverse experiences among the heterogeneous group of experts. Therefore, the findings should be interpreted as providing preliminary insights rather than an exhaustive account of all possible perspectives, but nonetheless offer valuable, context specific contributions that can inform future research, policy discussions, and potential implementation strategies in the region.

Including a broader range of stakeholders, such as decision-makers, pharmaceutical representatives, and patient organizations, could have provided a more comprehensive and nuanced understanding of the topic. The absence of patient and policy-maker perspectives limited the comprehensiveness of the analysis, particularly regarding acceptability, ethical considerations, reimbursement, and system-level feasibility, which are critical for real-world implementation, consequently limiting the policy implications of the findings.

Finally, efforts were made to ensure analytical rigor, transparency, and to mitigate potential biases. Nonetheless, the potential influence of the researchers’ affiliation, prior engagement with precision psychiatry, professional perspectives, and multilingual nature of the study should be acknowledged, as these factors may have shaped the framing of interview questions, interactions with participants, and interpretation of the data.

## Conclusion

This study offers novel insights into healthcare professionals’ perspectives on the value drivers and barriers to the adoption of precision psychiatry in LATAM, in which was found that, according to experts in the field, precision psychiatry holds significant promise for transforming mental health care by enhancing diagnostic accuracy, guiding treatments, and optimizing the use of resources, ultimately contributing to improved health outcomes and broader societal benefits. One of the key advantages of precision psychiatry, according to literature, lies in its integration of detailed clinical phenotyping with advances in neuroscience and data analytics [19, 59, 60]. This, however, remains an expectation, and empirical research in the coming years will determine whether precision psychiatry can fulfill its potential.

The implications of these perspectives are particularly relevant for MDD and its various phenotypes, given LATAM’s high burden of disease, prevalence of treatment-resistant cases, suicide risk, significant socioeconomic and quality-of-life impacts, and the challenges in diagnosing and treating MDD and its symptoms. The potential of precision psychiatry as a transformative approach [61], combined with the openness of healthcare providers and patients as identified by the experts, is a critical enabler for its adoption and development in LATAM.

The integration of precision psychiatry into clinical practice faces major perceived barriers. These include a limited understanding of what constitutes a precision approach in psychiatry, insufficient evidence of its clinical benefits and cost-effectiveness, inadequate provider knowledge, high implementation costs, and regulatory constraints. Additionally, existing challenges in MDD care in Latin America may further hinder adoption. These include under-resourced health systems, a shortage of trained healthcare professionals, limited access to medications, the low prioritization of mental health, persistent stigma, limited innovation culture, and the inherent complexity of diagnosing and treating psychiatric disorders.

Findings indicate that, from the experts’ perspective, the most important step to realize the potential of precision psychiatry is generating robust evidence across multiple dimensions, including clinical practice and decision-making, biomarker development and diagnostic accuracy, treatment response and effectiveness, prognosis and health outcomes, and cost-effectiveness. Achieving this requires increased research and training, stronger mental health policies, enhanced healthcare and information systems, and more effective regulatory and assessment processes, supported by coordinated efforts from diverse stakeholders to address both scientific and systemic barriers. Countries and decision makers could establish time-bound priorities based on these recommendations, for example, implementing training initiatives in the short term, strengthening data infrastructure and streamlining health technology assessment processes in the medium term, and developing cross-country, multicentric evidence-generation initiatives over the long term. Such efforts may contribute to the development of more robust explanatory models and refined taxonomies, ultimately supporting improved case formulation and more effective clinical decision making that can be adapted to diverse contexts, while allowing priorities to be tailored to each local setting.

The findings of this study contribute to a better understanding of the real-world perspectives and experiences of healthcare providers regarding the adoption of a precision approach in psychiatry. This approach presents a significant opportunity to address longstanding challenges in the diagnosis and treatment of MDD and other mental health disorders, with the potential for meaningful clinical and systemic improvements. Although the widespread implementation of precision psychiatry remains a long-term goal, this research in LATAM offers actionable recommendations that can serve as an initial roadmap for advancing the development and adoption of precision psychiatry in LATAM.

Future research efforts could explore the perspective of different stakeholders, assess the feasibility of implementation across different healthcare contexts, and develop context-specific strategies for adoption.

## Supplementary Information

Below is the link to the electronic supplementary material.

Supplementary Material 1

## Data Availability

The data that support the findings of this study cannot be made publicly available for confidentiality reasons. Data are however available from the corresponding author upon reasonable request.
