# Supplementary material for: Value drivers and barriers to the adoption of Precision Psychiatry in Latin America: a qualitative study
Source: BMC Psychiatry. 2026 Apr 3;26:406. doi: 10.1186/s12888-026-08038-5 (PMC13185392; doi:10.1186/s12888-026-08038-5)
Supplement: Supplementary file 1 — Supplementary Material 1 [file 12888_2026_8038_MOESM1_ESM.docx]

**SUPPLEMENTARY MATERIAL**

**Table of contents**

[Table S1. Overview of participants 2](#_Toc224037834)

[Table S2. Interview Guide 3](#_Toc224037835)

[Table S3. Summary of themes and subthemes, with expanded quotations 5](#_Toc224037836)

# Table S1. Overview of participants

| **Participant** | **Country** | **Professional Role** | **Years of experience** | **Primary Profile Category** | **Secondary Profiles** |
| --- | --- | --- | --- | --- | --- |
| Participant 1 | Argentina | Psychiatrist, High-level role in Medical Society | 15+ years | Health-care providers MDD | Mental health policy influencers |
| Participant 2 | Argentina | Professor, Psychiatrist, High-level role in Medical Society | 15+ years | Health-care providers MDD |  |
| Participant 3 | Brazil | Professor, Psychiatrist | 15+ years | Health-care providers MDD | Academic experts on precision psychiatry |
| Participant 4 | Brazil | Professor | 10+ years | Academic experts on precision psychiatry |  |
| Participant 5 | Canada | Professor, Psychiatrist | 15+ years | Academic experts on precision psychiatry | Health-care providers MDD |
| Participant 6 | Colombia | Psychiatrist, High-level role in Medical Society | 15+ years | Mental health policy influencers | Health-care providers MDD |
| Participant 7 | Germany | Professor, Psychiatrist | 15+ years | Academic experts on precision psychiatry | Health-care providers MDD |
| Participant 8 | Italy | Professor, Psychiatrist | 15+ years | Academic experts on precision psychiatry | Health-care providers MDD |
| Participant 9 | Mexico | Psychiatrist | 15+ years | Health-care providers MDD |  |
| Participant 10 | Mexico | Psychiatrist, High-level role in Medical Society | 15+ years | Health-care providers MDD |  |
| Participant 11 | Panama | Psychiatrist, High-level role in Medical Society | 15+ years | Mental health policy influencers | Health-care providers MDD |
| Participant 12 | Peru | Psychiatrist, National Health Council Representative | 15+ years | Mental health policy influencers | Health-care providers MDD |

# Table S2. Interview Guide

| **Question #** | **Question** |
| --- | --- |
| 1 | How would you describe the concept of precision medicine in psychiatry as understood within the mental health community in your country? |
| 2 | Are there any local initiatives or programs focused on precision medicine in psychiatry that you know of? |
| 3 | How familiar are local healthcare providers and decision-makers with precision medicine in psychiatry? |
| 4 | What sources of information or training about precision medicine in psychiatry are available locally? |
| 5 | Which sources of information would you look for to gain knowledge regarding this concept? |
| 6 | What is your perspective on the reliability of current diagnostic methods in psychiatry? |
| 7 | What do you consider targeted treatments in psychiatry, and how could they contribute to improved health outcomes? |
| 8 | In your experience, do patients understand what targeted approach means in psychiatry? |
| 9 | What strategies do you think would be most effective in educating policymakers and stakeholders about the potential health benefits of precision medicine in psychiatry? |
| 10 | What are the main challenges faced locally in diagnosing, treating, and following up MDD? |
| 11 | How do local healthcare providers address the heterogeneity of depression in their practice? |
| 12 | What are the primary barriers to accessing appropriate diagnosis and treatment for MDD in your community? |
| 13 | How do these barriers affect patient outcomes in your local context? |
| 14 | How do you think precision medicine in psychiatry could improve the treatment of MDD locally? |
| 15 | Which specific symptoms or patient characteristics do you believe could benefit most from a precision approach in the local context? |
| 16 | Which symptoms or characteristics do you think would be least receptive to a precision approach? |
| 17 | What do you think are the critical factors for successfully implementing precision medicine in psychiatry in your community? |
| 18 | How do you foresee precision medicine in psychiatry impacting patient care and health outcomes locally? |
| 19 | What are the economic and financial barriers to adopting precision medicine in psychiatry in the region? |
| 20 | What are the local nuances that need to be specifically included in local health technology assessment processes to help demonstrate the economic value of PP to local stakeholders? |
| 21 | What additional tools or approaches could be used to demonstrate the economic value of PP? |
| 22 | What regulatory and policy challenges do you foresee in implementing precision medicine in psychiatry locally? |
| 23 | What improvements to the regulatory and policy landscape would make the biggest gains in implementing precision medicine in psychiatry locally? |
| 24 | How can these challenges be addressed to facilitate the adoption of precision medicine in psychiatry? |
| 25 | What role do education and training of local healthcare providers play in the adoption of precision medicine in psychiatry? |
| 26 | What strategies would you recommend to improve awareness and knowledge of precision medicine in psychiatry among local psychiatrists and other mental health professionals? |
| 27 | What are the best tools or platforms to educate patients? |
| 28 | How can decision-making based on targeted treatments improve the selection of therapies, and what benefits would it bring to patients? |
| 29 | Do you think innovative treatments need to go beyond only biomarkers and genetics to be considered precision medicine in psychiatry? |
| 30 | Is there any treatment or intervention in your current practice that you would consider precision medicine in psychiatry? |
| 31 | In your practice, how do you view the potential of using targeted neurobiological pathways, even without clear biomarkers or genetic data, to guide treatment decisions for patients with MDD? How might this fit within your current clinical approach? |
| 32 | From your perspective, how could precision medicine in psychiatry, especially when following a symptom-focused approach, help reduce the treatment burden for patients by Improving the risk/benefit balance of therapies? |
| 33 | From your perspective, how could precision medicine in psychiatry, especially when focused on targeting specific neurobiological pathways, help reduce the treatment burden for patients by improving the risk/benefit balance of therapies? |
| 34 | What further advancements or tools do you think are necessary for innovative treatments without genetic or biomarker support to be fully integrated into precision medicine in psychiatry? How far do you think we are from realizing this vision? |
| 35 | What would be the challenges and opportunities in implementing a precision medicine in psychiatry approach with the treatments available today? |
| 36 | Who are the key local stakeholders that should be involved in studies and initiatives related to precision medicine in psychiatry? |
| 37 | How can we effectively engage these stakeholders to support the implementation of precision medicine in psychiatry in your community? |

# Table S3. Summary of themes and subthemes, with expanded quotations

| **Theme** | **Subtheme** | **Quotes** |
| --- | --- | --- |
| 1. Conceptualization and Understanding of Precision Psychiatry | Conceptualization of Precision Psychiatry | "Establishing a diagnosis or treatment based on specific biological characteristics or specific biomarkers." "To divide a group that is extremely heterogeneous into more homogeneous subgroups." "To treat the individual from their own point of view, taking into account the symptoms they have, the characteristics of the disease they seem to have, and their metabolism." "Precision medicine involves considering factors such as genetic variability, the patient’s lifestyle, and their environment, and, by taking all of these into account, designing a treatment plan." |
|  | Confusion around the concept of PP | "Precision medicine is confused with the term personalized medicine, which focuses more individually on each patient." "Targeted treatment is more closely linked to personalized, individualized medicine, which is similar but not identical to precision medicine. Targeted means looking at the specific characteristics of an individual and then choosing a treatment that is good for that particular patient." "The general idea among healthcare providers is that AI is involved and that you can predict something." |
|  | Familiarity of healthcare providers and patients | "Healthcare providers generally know what precision psychiatry is, but they lack detailed knowledge about specific methods, tools, or research findings, they have a general idea but not in-depth understanding." "Precision psychiatry is mostly discussed in academic circles or among particular interest groups, and even within that small expert community, there are different understandings of what it actually means." "Decision-makers tend to have more political affinity than technical expertise." "Patients know very little or almost nothing about it, though some family  members may have read about it." |
|  | Main  sources of information | "Some conferences and roundtables attract a lot of attention, but the professionals attending these talks often leave them somewhat frustrated because the content of the presentations is very limited." "Papers and scientific articles that talk about precision medicine." "All the information I read comes from the English-language literature." |
| 2. Challenges in Current Psychiatric Practice | Heterogeneity of psychiatric conditions | "The validity of the diagnosis is very low because it is a diagnosis based purely on signs and symptoms." "Unfortunately, I think that this is the main reason we are behind in precision medicine compared to other disciplines like oncology. The big problem is that our diagnoses are very heterogeneous. You can have two patients with major depressive disorder, with a major depressive episode, who do not share even a single symptom." "We do not have a way to describe the characteristics of a group of patients that differ greatly from another group or patient with the exact same diagnosis." "We are not precise enough when diagnosing patients, and in practice, we study patients who are very different from one another, even though they carry the exact same diagnosis." "Many end up using the same medications to treat very different cases. I even feel that the treatment for these cases is not very accurate, and many professionals end up leaving patients with just a partial response, not full remission, which should be our goal." |
|  | Reliability of diagnostic methods | "The reality is that our clinical evaluations still have some limitations; however, the use of diagnostic systems has an acceptable degree of reliability, which we rely on to establish our diagnoses." "The biggest problem for precision medicine in our field is that our classification system is not precise enough to support research and the discovery of biomarkers that could be useful for precision medicine." "For some diagnostic criteria it may be very easy to use the DSM-5 or the ICD-11 we are currently using, but for other pathologies, they are not so reliable, or if you follow the case over time, you can make mistakes in reaching a diagnosis at any given moment." "I believe that to this day, psychiatrists, even with major advances in multi-omics studies and some isolated neuroimaging studies, still do not have standardized studies for diagnoses that are both reliable and valid." |
|  | Awareness, stigma, and health-seeking behaviour | "Many patients do not seek treatment right away. They wait until it becomes something very serious, often due to stigma or certain prejudice against mental health. And when they do seek help, many drop out of treatment." "Stigma has decreased over the past decades, but the patient still resists seeing a psychiatrist. Strangely enough, they prefer to be treated by a non-specialist." "Stigma not only among the general population, but also among colleagues from other specialties. Sometimes the psychologist also has their own stigma and does not refer the case for psychiatric care." "Some health insurance companies still do not cover psychiatric issues, including depression." |
|  | Availability and Knowledge  of Healthcare providers | "The system does not have enough psychiatrists to handle all psychiatric cases. As a result, patients end up being treated by other professionals: non-specialists, general practitioners, neurologists." "Validity in primary care by healthcare professionals is relatively low. They either do not recognize depression or treat it too long without referring appropriately." "Psychologists are part of the diagnostic process, but while they can offer therapy, they cannot prescribe medications, which are more often needed for moderate to severe cases." "The wait time for a psychiatric appointment can be very long, around 3 or 4 months, starting from the initial primary care visit, through referral." |
|  | Access to medicines | "Psychotropic medications are expensive and hard to access. It is difficult to find all the prescribed medicines in a single pharmacy." "The list of medications approved by the Ministry of Health for depressive disorder is very limited. Many medications are not yet approved due to bureaucratic hurdles required for registration." "The main issue is treatment: accessing medications is very difficult. The health system may provide one drug, then switch it for another, and then another; there’s no continuity." "There’s a lack of available treatment options, almost no non-pharmacological measures are offered, and pharmacological ones are very limited, often with significant side effects and safety concerns." |
|  | Impact on outcomes | "Delays in starting treatment mean patients experience more depressive episodes and more hospitalizations due to untreated depression. These delays affect quality of life, the ability to function, return to work, and maintain family and social relationships, and many outcomes worsen over time." "Patients feel they have recovered, stop treatment, and fall into repeated relapses and treatment-resistant depression." "Rising rates of treatment-resistant depression and inadequate treatment responses make it hard to tell if a patient truly has resistant depression or if their depression is simply undertreated." "In general medical training, mental health is not well covered, so doctors graduate with gaps in diagnosing and managing these conditions. By the time patients reach specialists, they often have had treatment failures or poor adherence due to a lack of explanation and psychoeducation." |
| 3. Value and Drivers of Precision Psychiatry | Support Clinical Decision-making | "It would dramatically change the approach to mental illness. It would be more proactive, more preventive, and would likely reduce morbidity and mortality associated with mental illness in the long term." "A biological individualization of these patients into more homogeneous biological subgroups will certainly enable more effective treatments with fewer side effects." "But if we find a biomarker, if we find any major indicator that guides us in choosing a certain treatment, knowing that this medication is better for these symptoms and that one for others, I think it will be easy to adopt." "Psychiatrists believe we treat based on diagnoses, but actually, our treatments are symptomatic. Improving symptomatic approaches could be very interesting because it could reduce disease burden, provide relief, and greatly improve quality of life." "That is, we might need to slightly modify classifications, because right now classifications include all symptoms but do not subdivide depression into symptom-based subtypes tied to affected neural circuits." "It would increase the chance of benefit and reduce the risk of side effects and unwanted safety hazards." "It would also give doctors more confidence when making difficult decisions." |
|  | Clinical benefits | "The goal is to identify patients who are likely to benefit from these therapies earlier. Early identification and effective treatment would improve outcomes, reduce complications, and enhance efficacy while minimizing exposure to side effects." "This would also reduce the risk of side effects and minimize patients’ exposure to ineffective treatments." "I believe it would help significantly if the right information is available, such as previous treatments and comorbidities. Combined with genetic data, this would greatly enhance not only diagnosis but also prognosis and treatment planning." "By treating the patient in a personalized way and focusing on their specific needs, their depressive condition and quality of life will likely improve." "By using transdiagnostic approaches, big data, and data science, I believe we will see major improvements in prevention, diagnosis, and treatment." In terms of treatment selection, it will help us choose the best option instead of relying on trial-and-error strategies." |
|  | Health-system and societal benefits | "It would help rapidly identify complex cases that need to be seen by the few specialists we have and optimize the use of scarce resources." "Following the problem or moving toward an accurate diagnosis would bring many benefits, helping prevent relapses, reduce or avoid hospitalizations, and allow patients to function in society without disruption to daily life." "In the long term, this would reduce national costs because we’d have patients who visit clinics less, spend less on healthcare, are more productive, and whose families are also more productive." "Over time, from an economic and health outcome perspective, expenses should improve. A stable patient means fewer consultations, fewer hospitalizations, and increased productivity. So overall, the long-term impact would be positive." |
|  | Benefits of Precision Psychiatry for MDD | "I think the most severe cases should be prioritized, such as depression with psychotic features, suicide risk, and extreme self-neglect, because these indicate greater illness severity." "Some symptoms respond very well to certain medications and very poorly to others. For instance, insomnia: some medications treat it effectively, while others actually cause it. The same goes for hypersomnia, anxiety, agitation versus psychomotor retardation. These are the symptoms that can benefit the most, because there is significant variability in how medications work across them, so we need to understand the drivers." "For example, the anhedonia cluster is very hard to treat, so it would be a challenge for a company to develop a drug that targets it specifically. Other symptom clusters like sleep issues, which are integral to depression, could also be targeted." "There are some that are especially hard to treat, like anhedonia and cognitive symptoms. These would benefit most from precision approaches." |
|  | Receptiveness by healthcare providers and patients | "I think that the main advantage is that these professionals are very interested and receptive to this kind of approach. In other words, if we have even minimal scientific evidence to guide professionals toward the best treatment for each patient, they are very open to it, and so are the patients." |
|  | Pathways to Establish Precision Psychiatry | "All the specific tools of precision medicine, whether biomarkers, genetics, or others, are currently tied to existing classifications. But these classifications are limiting. We do not need to abandon them but rather complement them." "It is obvious that biomarkers and genetic markers are important, but only if they result in a product that actually improves treatment and the patient’s quality of life. If that does not happen, they are of no use." "We should not just look at individual genes or biomarkers, we should examine systems and neurobiological pathways, such as inflammation or neurotrophic factor pathways. If we know these are dysregulated, it can guide treatment." "Neurophysiology, neuroimaging, and clinimetrics should also be implemented, along with data generated from electronic health records and digital lifestyle tracking. When this is all included, the algorithm becomes incredibly powerful." "Focusing only on genetics, biomarkers, and omics is too narrow a view. Social, psychological, behavioral, and environmental factors also matter and must be considered." |
| 4. Barriers and Challenges of Precision Psychiatry | Supporting Evidence | “They need something concrete that shows how precision medicine saves time, treatment resources, money, and time doctors and nurses spend with patients. In other words, we need to link the results of precision medicine to clear resource savings.” “This is a very important issue. On the one hand, we want to educate policymakers and stakeholders because, as clinicians, we believe in the concept of precision psychiatry. But on the other hand, we lack the evidence.” “We do not have proof that precision psychiatry actually works in practice.” “We need clinical trials that assign patients to different treatments in a randomized fashion and then analyze whether we can identify markers that show which patients benefit more from one treatment versus another. Designing these studies will be a challenge, but we could begin by analyzing real-world data retrospectively to get an initial idea.” |
|  | Costs | “The cost of medications is a major barrier, they are not affordable. The public health limits the number and type of available drugs, and even in the private sector, most patients cannot afford many of them due to financial constraints.” “Experts consistently emphasized that cost is a primary obstacle to implementing precision medicine. While the techniques themselves are not necessarily invasive, developing and applying precision approaches, such as building models or using genetic tests, requires significant investment. These methods demand many hours of work from both domain specialists and experts in artificial intelligence and computational sciences.” “Ultimately, if patients are expected to pay out of pocket, many simply will not be able to. Precision medicine must become financially accessible to the end user, otherwise, it cannot be widely implemented, especially without a payer system in place. These treatments will likely be costly at first due to the complex integration of translational medicine, clinical care, and engineering.” |
|  | Investments in Health | "Even before the pandemic, the budget for mental health had been increasing. So, from a financial standpoint, money does exist; the problem lies in management." "There is very little investment in mental health, and I believe that is a major problem. Health spending is around 3% of the GDP, and investment in mental health is even smaller; that is the main issue." "The situation is particularly difficult because there is not currently a substantial budget for health and research. Much of the work is carried out by research teams who secure limited funding, which makes advancing precision psychiatry quite difficult." |
|  | Regulatory Landscape | “The public system looks directly at the cost of treatment. It has a great deal of difficulty conducting pharmacoeconomic studies. They only consider the immediate and direct costs. They do not consider the long-term impact this might have.” “If professionals are well trained and confident in their approach, they use treatments off-label, that is our day-to-day reality in psychiatry.”  “The issue is if the treatments are very expensive, we need to demonstrate that they don’t cost more than the savings they will generate.” “In terms of regulatory and policy processes, the big issue is that it takes a  long time for anything to be approved.” “There is always a strict protocol. This could be a limiting factor, especially with treatments more than diagnostic tests. All medications require a series of studies before they can be introduced into the public system. It is another set of bureaucratic steps that must be followed.”  “The steps take time because the people involved in these regulatory processes are not very familiar with concepts” |
|  | Mental Health Prioritization | “It comes down to public policy, because usually the policymakers and decision-makers are not the most up-to-date people in the field. They are typically not professionals who practice the specialty.” “I think that often regulators and policymakers, when evaluating a new treatment, rely on existing treatment guidelines, which are based on studies that use conventional, not precision psychiatry.” “Public policies often marginalize people with mental illness. Even though mental health gained some prominence after the pandemic and there is renewed attention to primary health care, there is still a long way to go.” “There’s still stigma, although it is slowly improving, where psychiatric patients are seen as a burden, not worth investing in.”  “In terms of public health strategies, we have very few targeted strategies for major depression treatment, almost nothing focused on this population.” |
|  | Knowledge Among Health Care Providers | “The other major challenge is the lack of training and lack of access to quality information for the vast majority of specialists, who are not researchers and are not based in universities. Research is not part of their daily routine.” “In each of the multi-omics areas, for example, many colleagues do not even know what genomics is.” |
|  | Least Receptive Cases of MDD | “These would be symptoms more related to personality disorders. For example, a histrionic personality trait: those symptoms are much more linked to non-biological aspects, more psychological aspects of the illness. So, for personality traits, I think there is much less potential for precision psychiatry.” “Because social characteristics are also very important in precision psychiatry, and I think we need to include them. But on the other hand, they are hard to change. So social circumstances are difficult to modify, but I think it would be a mistake to exclude them.” |
|  | Ethical considerations | “This type of research requires equipment like functional MRI or functional PET. Some may also require exposure to contrast material, which can be somewhat dangerous. They may require exposure to radioligands.” “Is there a risk that algorithms make mistakes? Yes, we know that AI models make diagnostic errors. Mistakes happen, so there will be a need for professional oversight.” “There’s also everything related to privacy, because behind all this, even if it sounds very nice, our data will be used: for what? That hasn’t been addressed yet.” |
| 5. Current and Future Status of  Precision Psychiatry | Existing Experiences and Initiatives | “Based on the patient’s history and analysis of environmental factors, we can better guide treatment in psychiatry. This is a start.” “Yes, we have off-label treatments today. For example, based on some studies and empirically, if a patient has treatment-resistant symptoms and hypercortisolemia, using a cortisol synthesis inhibitor tends to improve the patient’s response.” “Cognitive behavioral therapy, for example, for insomnia, is precision medicine. It is not something we provide to all depression patients, but to some where sleep is clearly seriously affected.” “Yes, I have easy access to pharmacoeconomic and pharmacogenomic tests. We have the Neurophysiology Department, the Clinimetrics Department, even treatments using virtual reality, some psychotherapy subtypes, and neuromodulation in different modalities.” “I have already requested pharmacogenetic markers. I frequently request neuroimaging.” |
|  | Time for Realization | “We are still far, very far. The main reason is that studies and clinical trials continue to focus on global diagnoses without stratification. So, investment will need to go into stratified studies of the pathology.” “And yes, we are still quite far. We need to go beyond genetics and biomarkers.” “At the moment, I am not aware of any very strong biomarker that is widely usable or accepted.” “However, from a bioethical standpoint, I think we are still far from being able to access and integrate such a vast amount of information without invading people’s privacy.” “There will be no impact in the next three to four years; it is still aspirational.” |
| 6. Recommendations | Generate Evidence | "Critical factors include first providing evidence. We must generate and demonstrate that precision psychiatry works and offers benefits compared to conventional treatment. This requires a major effort to generate that evidence." "Showing that using precision medicine saves money, time, and resources is essential. That is what most policymakers and stakeholders look for, and it’s understandable. While it’s important to prove that precision medicine is helpful, what truly facilitates its adoption is demonstrating savings of money, time, and resources." "Qualitative data can later be transformed into more quantitative analysis, but without that data, decision-makers, who are often not even doctors, will not understand the benefits of being more targeted and specific." "And we should combine pharmacoeconomics with quality of life studies: What does it mean to have a stable patient, not just for hospital metrics, but also in terms of productivity, reduced hospital time, family wellbeing, and community impact?" "There are many imported pharmacoeconomic studies, but very few from Latin America. I think collecting local data in our countries would be very helpful. Most pharmacoeconomic studies I’ve seen come from abroad." |
|  | Expand Awareness and Training in Precision Psychiatry | "Critical factors for successful implementation include knowledge, as education and training of local healthcare providers are crucial; they are the gatekeepers for precision psychiatry’s success and commercialization." "We can have public policies, guidelines, and all the tools, but without continuing education and training, and this training cannot be restricted to universities, it will not be effective." "Webinars, symposiums, short papers, or brochures can be very helpful." "Topics should be modern, dynamic, and institutionalized with the support of scientific societies and governments." "More work is needed in medical and psychology schools to incorporate precision medicine into curricula." "Scientific dissemination in conferences is important, but leadership is also needed, perhaps by a specialized mental health center with financial support." |
|  | Improve Health Technology Assessment Processes | "Basically, what they need to know is how these new techniques can affect their costs. Costs not only in terms of money, but also in terms of time that doctors have to spend with a patient before the patient feels well; the time the patient has to spend in the hospital before they improve; the time the patient must stay off work, which is also a cost to society." "I think the initial improvement needed is that when a new drug is planned to come to market, companies should first talk to regulatory bodies and get advice." "We must include health technology assessment processes with not only clinical but also economic studies that demonstrate impact. I emphasize not only the direct healthcare costs of treating patients with depression but also indirect costs." "There needs to be improvement in recognizing cost-effectiveness by regulators and policymakers, recognizing the cost advantages of using precision psychiatry." "I think that will be a significant question: will the research methodologies of regulatory trials adopt precision psychiatry methods, and how will regulators respond? We don’t know yet."  “For certain biological markers that work, how the regulatory bodies will respond is uncertain: whether they will treat this as a new type of design and consider whether it can be compared to previous designs with earlier drugs.” |
|  | Increase Funding for Mental Health and Research | "We also need to be honest and admit that we do not yet know the full potential benefits of precision psychiatry. We assume there are some advantages, but scientifically, this is still uncertain. We definitely need more funding programs for research in precision psychiatry." "I believe part of the issue is increasing the budget dedicated to mental health. I also think there should be some improvements in the training and education of psychiatrists." |
|  | Strengthen Mental Health Policies | "We need changes at the level of public mental health policies. I believe the critical factor for implementation is to have a strong, organized proposal or team and political will to make it happen." "One policy could be to include this topic within government educational programs for psychiatrists and other healthcare providers." "It is a matter of public policy because usually, the policymakers and decision-makers in public health are not the most updated professionals in the field. They are generally not practitioners of the specialty." |
|  | Engage Stakeholders | "I think we need to not only look at researchers, but also include industry partners, policymakers, and stakeholders such as patient organizations. So, it must be a broad interdisciplinary approach. Bringing them together is a critical factor for generating evidence and planning for implementation, fostering joint understanding. This requires closer communication between industry, researchers in precision psychiatry, and policymakers." "Patients, doctors, and mental health professionals, together with support groups and scientific societies, are key actors who must be involved in implementing this, especially when aiming to influence mental health public policies. Encouraging the formation of these support groups and fostering alliances that ensure patients and families feel heard is vital." "There should be collaborations between government groups and research institutions that share a common goal of understanding psychiatric disorders and working together to improve the system. Research teams should include not only doctors and psychiatrists, but also neurobiologists, neurologists, imaging specialists, and engineers who can collaborate in an integrated way." |
|  | Enhance Healthcare Service Provision | "We need to expand the system's capacity to serve patients who require specialists, both in terms of available medications and access to specialized professionals." "We may need a different approach, where not everyone with MDD first goes through primary care, then secondary, and then tertiary care. We need to build something that differentiates cases much earlier." "There are opportunities if we think beyond stigma and people begin to see us more as healthcare providers, like any other medical professional, and finally recognize psychiatry as a specialty to consult in complex cases, even from general, first-contact mental health providers." |
|  | Improve Integration and Management of Healthcare Data Systems | "The information I can access about a patient is only available to the patient and me in my medical record. But if the patient sees another colleague, that information probably will not be accessible or useful for improving care." "I think there needs to be alignment with local medical records, digital records, and electronic records. We are talking about different healthcare systems, so to demonstrate impact, we’ll need large, integrated population data. That will be a challenge in some jurisdictions." "I believe some artificial intelligence applications have recently been approved for clinical use and are already integrated into electronic health records. People are no longer as resistant to this." |
|  | Leverage International Experience | “In the latest international updates of guidelines, precision psychiatry is already being implemented.” “I would suggest presenting external experiences from countries that have already made progress in precision medicine, what they have achieved, and how we could apply that to our population. Of course, this should consider the genetic characteristics of our population, which are very diverse. We would need to apply or adapt their advancements accordingly. “ |
